# Predicting antioxidant activity of wood vinegar using color and spectrophotometric parameters

**DOI:** 10.1016/j.mex.2020.100783

**Published:** 2020-01-07

**Authors:** Krittaya Petchpoung, Siriwan Soiklom, Wipada Siri-anusornsak, Nathawat Khlangsap, Anucha Tara, Thanapoom Maneeboon

**Affiliations:** a Scientific Equipment and Research Division, Kasetsart University Research and Development Institute, Kasetsart University, Bangkok, 10900, Thailand; b Center for Research Stations and Demonstrate Forests, Faculty of Forestry, Kasetsart University, Bangkok, 10900, Thailand; c Trad Agroforestry Research and Training Station, Faculty of Forestry, Kasetsart University, Trad Province, 23000, Thailand

**Keywords:** Simple, rapid and no chemical reaction involved for antioxidant activity prediction by color and spectrophotometric parameters, Predicting model, Simple, Rapid, No chemical reaction involved

## Abstract

Wood vinegar can be produced from many types of raw materials using different pyrolysis methods resulting in potentially different antioxidant activity. Therefore, this study developed a rapid method to predict the antioxidant activity of wood vinegar based on color using the CIELAB system and spectrophotometric parameters. The 2,2-diphenyl-1-picrylhydrazyl (DPPH) radical scavenging activity and ferric reducing antioxidant power (FRAP) exhibited significant correlations with the L* and b* values of the color parameters and the UV absorbance polyphenol index (I280), European Brewing Convention (EBC) and Institute of Brewing (IOB) color units, color intensity and Linner Hue index of the spectrophotometric parameters. As a result, DPPH radical scavenging activity and FRAP could be predicted by measuring color and spectrophotometric parameters. Overall, this study provide a simple, rapid and no chemical reaction involved method to predict antioxidant activity. Furthermore, models with a set of spectrophotometric parameters could be used to predict antioxidant activities.

• Whole new method for predicting wood vinegar antioxidant activity was developed.
• The method was models developed by using color and spectrophotometric parameters used in beverage industry.
• The models were simple, rapid and involved no chemical reaction.

## Graphical abstract

**Figure:**
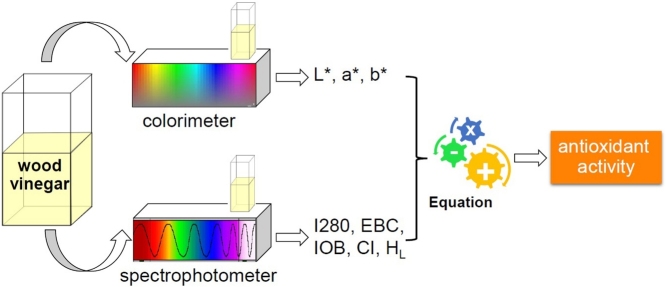

Specification Table

**Table:** 

Subject Area:	Chemistry
More specific subject area:	wood vinegar
Method name:	Simple, rapid and no chemical reaction involved for antioxidant activity prediction by color and spectrophotometric parameters.
Name and reference of original method:	L.R., Bishop. Proposed revision of the Lovibond “52 series” of glass slides for the measurement of the colour of worts and beers, J. Inst. Brew. (1950) 373–382.
Resource availability:	N/A

## Method details

### Background

Wood vinegar or pyroligneous acid is one of the major by-products of charcoal production. It contains several types of compounds, including alcohols, aldehydes, esters, furans, ketones, organic acids, phenols and pyrans [[1], [2], [3]]. A number of wood or agricultural residues, such as eucalyptus, oak, rubber, bamboo, mangrove, cotton stalk, almond shell, rice hull and straw have been used in the production of wood vinegar [4]. Furthermore, a diverse range of pyrolysis methods to produce wood vinegar have been reported [[5], [6], [7]]. Wood vinegar has shown ability as a fungicide and insecticide [8], an antidermatitis agent [9], a rubber coagulant [10] and an antioxidative agent [11].

The color of wood vinegar ranges from reddish- or yellowish-brown to deep dark brown. Its color range is comparable to some types of beer, caramel, tea and wine and colorimetric and spectrophotometric properties have been used for a long time to determine and classify these beverage products. In 1883, a series of 52 color discs was proposed by Lovibond as a visual method for measuring the color in beer. Later, in 1950, the method was revised by Bishop [12]. Afterwards, the EBC adopted the revised method for the analysis of beer color. Determination of absorbance using a spectrophotometer is also a recommended method of the EBC and the IOB [13]. Apart from spectral absorbance, color as a tristimulus value and CIELAB are also applied as measuring methods [14]. Methods similar to that for beer color analysis have been applied to determine caramel and wine color as well. Furthermore, the direct measurement at 280 nm has been used as a simple method for measuring the polyphenols present in wine [15,16]. In addition, the association has been reported of the antioxidant activity and polyphenol content of beer, caramel-containing soft drinks, tea and wine with color or spectrophotometric parameters [[17], [18], [19], [20]].

To our knowledge, there has been no reported investigation to date on the estimation of antioxidant activity using the color and spectrophotometric parameters of wood vinegar. The objective of this study was to develop a rapid method to predict antioxidant activity using both the color based on the CIELAB system and the spectrophotometric parameters of wood vinegar.

### Materials and methods

#### Wood vinegar samples

The 88 wood vinegar samples analyzed in this study were collected in 2017 and 2018 from several provinces in Thailand including Bangkok, Nakhon Ratchasima, Ratchaburi, Tak, Trad and Ubon Ratchathani.

#### Analysis of color and spectrophotometric parameters

The colors of the wood vinegar samples were determined in terms of the CIELAB parameters (L*, a* and b*) using a colorimeter (HunterLab, Ultrascan Pro, USA) with illuminant D_65_, 10° observation and a 10 mm path length cell. L* defines lightness, where a low L* value (minimum 0) indicates dark and a high L* value (maximum 100) indicates light. Red/green and yellow/blue colors are denoted by a* and b* values, respectively. An increase in the a* value depicts a shift toward red while a decrease in a* value depicts a shift toward green. Similarly increasing and decreasing the b* value relates to increasing yellowness and blueness, respectively.

Appropriately diluted wood vinegar which resulted in range of 0.1–1.0 absorbance units at wavelength of 280, 420, 430, 510, 520, 530, 610 and 620 nm was measured by a UV–vis spectrophotometer with a 10 mm path length cell (Shimadzu, UV-1800, Japan).

According to the EBC Recommended Method, absorbance at 430 nm was measured and multiplied by 25 and a dilution factor, to give a color value in terms of the EBC color units [21].

IOB color units was determined from the absorbance at 530 nm multiplied by 1,000 and a dilution factor [13].

According to the wine color intensity method, the color intensity (CI) was calculated as the sum of absorbance multiplied with dilution factor at 420 nm, 520 nm and 620 nm [22].

In consonance with caramel color tone measuring methods, the Linner Hue index (H_L_) of wood vinegar was determined as 10log(A510/A610) where A510 and A610 are the absorbance multiplied with dilution factor at 510 and 610 nm, respectively [23].

The UV absorbance polyphenol index (I280) was calculated from the absorbance at 280 nm multiplied by a dilution factor [16].

### Antioxidant activity measurement

#### DPPH radical scavenging activity assay

DPPH radical scavenging activity was determined as described previously [24]. A aliquot of 100 μL of each appropriately diluted sample was reacted with 2.9 mL of 0.06 mmol/L DPPH reagent. The absorbance at 517 nm was measured after incubation for 60 min in the dark. The DPPH radical scavenging activity was expressed as milligrams gallic acid equivalent (GAE)/L.

#### FRAP assay

The ferric reducing antioxidant power of samples was measured using the method described by Benzie and Strain [25] with slight modifications. In brief, FRAP reagent was freshly prepared by mixing 1 part of 10 mmol/L 2,4,6-tri(2-pyridyl)-*s*-triazine, 1 part of 20 mmol/L ferric chloride and 10 parts of 300 mmol/L sodium acetate buffer (pH 3.6). Then, 100 μL of each appropriately diluted sample was added to 3 mL of FRAP reagent. The absorbance at 593 nm was recorded after incubation at 37 °C for 4 min. Results were calculated in terms of milligrams of trolox equivalent (TE)/L.

### Statistical analysis

All samples were analyzed in triplicate. Data were presented as mean ± standard deviation (SD). Correlation analysis among color, spectrophotometric and antioxidant activity was performed based on Pearson's correlation test. Linear regression analysis using the enter method was performed to obtain equations for estimating antioxidant activity. A p-value less than 0.05 was considered significant. All statistical tests were carried out using SPSS for Windows.

## Results and discussion

The color, spectrophotometric and antioxidant properties of 88 wood vinegar samples are shown in Table 1. All wood vinegar samples had positive values for a* and b*, as was expected since wood vinegar commonly being a reddish or yellowish color. However, there was wide range in lightness from very dark (L* = 1.09) to notably light (L* = 87.75).

**Table 1:** Color, spectrophotometric and antioxidant properties of wood vinegars.

Parameter	Mean ± SD	Range in values
L*	60.72 ± 20.58	1.09–87.75
a*	28.52 ± 11.96	1.63–50.93
b*	85.54 ± 25.38	1.72–103.77
I280	331.91 ± 204.35	93.83–995.62
EBC	159.18 ± 146.36	27.36–826.03
IOB	1,604.15 ± 2,430.41	147.25–11,243.70
CI	9.83 ± 10.29	1.54–54.61
H_L_	8.26 ± 1.92	2.71–14.27
DPPH (mg GAE/L)	3,136.67 ± 1,616.85	886.73–8,688.58
FRAP (mg TE/L)	8,912.89 ± 7,591.18	1,667.45– 44,241.08

Significant correlations were observed between the DPPH radical scavenging activity and all parameters, though a* showed a weak correlation. Beside a*, the FRAP was also significantly correlated with all parameters. Increasing values of I280, EBC, IOB and CI exhibited higher DPPH radical scavenging activity and FRAP. On the other hand, higher values of L*, b* and HL resulted in lower DPPH radical scavenging activity and FRAP as shown in Table 2.

**Table 2:** Correlation coefficients among antioxidant activity, color and spectrophotometric parameters.

	DPPH	FRAP	L*	a*	b*	I280	EBC	IOB	CI
FRAP	0.874**								
L*	−0.750**	−0.839**							
a*	0.193**	0.043	−0.318**						
b*	−0.641**	−0.821**	0.857**	0.128*					
I280	0.844**	0.785**	−0.765**	0.368**	−0.639**				
EBC	0.810**	0.723**	−0.763**	0.357**	−0.649**	0.892**			
IOB	0.847**	0.867**	−0.829**	0.073	−0.819**	0.806**	0.868**		
CI	0.866**	0.809**	−0.812**	0.253**	−0.732**	0.887**	0.982**	0.932**	
H_L_	−0.639**	−0.743**	0.749**	−0.073	0.751**	−0.585**	−0.610**	−0.728**	−0.675**

*and** = significant at 0.05 and 0.01 probability levels, respectively.

The regression equations for prediction of the antioxidant from wood vinegar are presented in Table 3 based on the coefficient of determination (R^2^) and root mean square error (RMSE) for the predictions.

**Table 3:** Regression equations for prediction of antioxidant activity.

Antioxidant activity	Equation	Parameter	R^2^	RMSE
DPPH	= −79.57L* − 21.65a* + 15.75b* + 7,254.18	Color	0.570	1,065.77
	=4.06i280 − 21.80EBC − 0.22IOB + 419.30CI − 16.16H_L_ + 1,355.74	Spectrophotometric	0.856	618.98
	= −15.43L* −6.37a* + 26.31b* + 4.11i280 − 23.77EBC − 0.12IOB + 441.08CI − 42.88H_L_ + 663.38	Both	0.881	565.88
FRAP	= −328.13L* −149.95a* − 8.64b* + 33,842.37	Color	0.760	3,738.05
	=19.74i280 − 107.70EBC + 0.18IOB + 1,633.38CI − 638.63H_L_ + 8,440.27	Spectrophotometric	0.880	2,653.85
	= −103.09L* −47.19a* + 6.01b* + 17.91i280 − 92.93EBC − 0.06IOB + 1,403.43CI −375.45H_L_ + 14,266.87	Both	0.893	2,525.58

The R^2^ values for the three-color parameter regression equation for DPPH radical scavenging activity and FRAP were 0.570 and 0.760, respectively. The R^2^ values of the equations with the five spectrophotometric parameters were higher than for the equations with three color parameters. In spite of that, the predictive performance was better when using a combination of all eight of the color and spectrophotometric parameters. However, the power of prediction for FRAP was slightly lower than for DPPH radical scavenging activity.

**Fig. 1:**
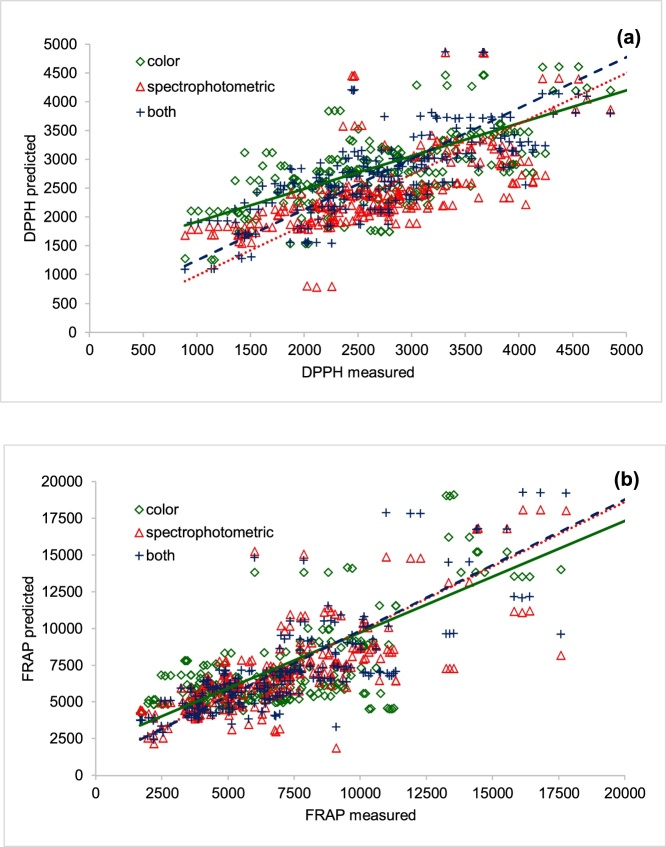
Comparison of measured and predicted antioxidant activity: (a) DPPH (b) FRAP.

The predictions of antioxidant activity were compared with the measurement (Fig. 1). Solid, dot and dashed lines indicate the measurement equal to the prediction by color, spectrophotometric and both set of parameters, respectively. It was clear that predictions using the five spectrophotometric parameters were similar in trend and quality to the equation with all eight parameters, especially for FRAP prediction. The results presented in Table 3 and Fig. 1 also suggested that color parameters alone were not sufficient to provide a good prediction.

Therefore, the proposed predicting models for DPPH radical scavenging activity and FRAP are Eqs. (1) and (2), respectively.

(1) DPPH = -15.43L* -6.37a* + 26.31b* + 4.11i280 - 23.77EBC - 0.12IOB + 441.08CI - 42.88H_L_ + 663.38

(2) FRAP = -103.09L* -47.19a* + 6.01b* + 17.91i280 - 92.93EBC - 0.06IOB + 1,403.43CI -375.45H_L_ + 14,266.87

This study also proposed the alternative predicting models based on only a set of spectrophotometric parameters as Eqs. (3) and (4) for DPPH radical scavenging activity and FRAP, respectively.

(3) DPPH = 4.06i280 - 21.80EBC - 0.22IOB + 419.30CI - 16.16H_L_ + 1,355.74

(4) FRAP = 19.74i280 - 107.70EBC + 0.18IOB + 1,633.38CI - 638.63H_L_ + 8,440.27

## Conclusion

This study showed that lightness, yellowness, UV absorbance polyphenol index, EBC and IOB color units and the Linner Hue index were significantly correlated with the DPPH radical scavenging activity and FRAP of wood vinegar. Wood vinegar that was darker and less yellow was likely to have higher antioxidant activity. Furthermore, the study showed that wood vinegar antioxidant activity could be predicted by measuring the color and spectrophotometric parameters used in the beverages industry. The developed models of prediction from the study were inexpensive, simple and rapid and involved no chemical reaction. It is also environmentally friendly method and minimum waste generation. Despite not being as good as models involving both sets of parameters, the model based on the spectrophotometric parameters alone could be used for estimating antioxidant activity as well.

## Declaration of Competing Interest

The authors declare that they have no known competing financial interests or personal relationships that could have appeared to influence the work reported in this paper.
